# Dynamic Contour Irregularities in Implant-Based Breast Reconstruction: Long-Term Aesthetic Outcomes After Direct-to-Implant Versus Tissue Expander Techniques

**DOI:** 10.3390/jcm14238452

**Published:** 2025-11-28

**Authors:** Theresia Stigger, Selina Neurauter, Barbara Del Frari, Katharina Spechtler, Maria Emilia Casari, Hannes Neuwirt, Ines Schoberleitner, Daniel Egle, Christine Brunner, Dolores Wolfram

**Affiliations:** 1 Department of Plastic, Reconstructive and Aesthetic Surgery, Medical University of Innsbruck, Anichstrasse 35, 6020 Innsbruck, Austria; theresia.stigger@i-med.ac.at (T.S.); selina.neurauter@tirol-kliniken.at (S.N.); barbara.del-frari@i-med.ac.at (B.D.F.); katharina.spechtler@i-med.ac.at (K.S.); maria.casari@tirol-kliniken.at (M.E.C.); ines.schoberleitner@i-med.ac.at (I.S.); 2 Department of Internal Medicine IV, Medical University of Innsbruck, Anichstrasse 35, 6020 Innsbruck, Austria; hannes.neuwirt@i-med.ac.at; 3 Department of Gynecology and Obstetrics, Medical University of Innsbruck, Anichstrasse 35, 6020 Innsbruck, Austria; daniel.egle@tirol-kliniken.at (D.E.); c.brunner@i-med.ac.at (C.B.)

**Keywords:** breast, breast reconstruction, implant-based breast reconstruction, direct-to-implant, tissue expander, animation deformity, dynamic implant contour irregularities

## Abstract

**Background**: Animation deformity (AD) is a well-known complication of submuscular implant placement. However, dynamic contour irregularities have also been reported in prepectoral reconstructions, but their mechanisms and classification remain unclear. **Methods**: In this prospective, single-center study, 67 patients underwent either direct-to-implant (DTI, n = 33) or tissue expander (TE, n = 34) reconstruction. Among these, 56 patients had subpectoral and 11 patients had prepectoral implant placement. Patient-reported outcome measures (PROMs) were assessed using the BREAST-Q. Aesthetic outcomes were rated by four plastic surgeons using the Breast Aesthetic Scale. Breast animation deformity was evaluated clinically and photographically by an expert panel using two distinct classification systems. **Results**: The mean follow-up period was 78 (±45) months. No significant differences were found between DTI and TE groups in BREAST-Q scores or overall aesthetic outcomes. Bilateral reconstructions showed significantly higher aesthetic ratings (*p* = 0.001). AD was observed in 61.2% of patients, with significantly higher prevalence in the DTI group (*p* = 0.02). Six patients with prepectoral reconstruction demonstrated dynamic contour irregularities distinct from classical AD. A new definition for this phenomenon is proposed. **Conclusions**: DTI and TE reconstructions achieve comparable long-term patient satisfaction and aesthetic outcomes. The observed cases of dynamic implant contour irregularities (DICI) in prepectoral implant placement underscore the need for a more nuanced classification of contour irregularities in implant-based breast reconstruction beyond the established concept of AD.

## 1. Introduction

Breast reconstruction is a key component of comprehensive breast cancer care. As survival rates rise and more patients opt for reconstruction following mastectomy, the emphasis has increasingly shifted towards optimizing aesthetic outcomes and quality of life [1,2,3,4,5]. Among implant-based reconstruction techniques, two dominant approaches have emerged: direct-to-implant (DTI) versus tissue expander (TE) reconstruction, each offering distinct advantages and challenges [6]. In the single-stage DTI approach, the definitive implant is placed immediately at the time of the mastectomy, while the two-stage TE technique involves initial placement of a tissue expander, followed by a second surgery to insert the final implant [7,8]. Based on a national U.S. database, TE reconstruction remains the more common technique (83%) compared to DTI reconstruction (17%). However, DTI reconstructions have shown a steady annual increase nationwide [9]. A recent trend indicates an increasing preference for prepectoral over subpectoral implant placement, primarily due to the lower incidence of animation deformity (AD) [10,11].

Previous studies focused predominantly on comparing these techniques based on complication and revision rates [12,13,14]. While some studies report similar complication profiles between DTI and TE approaches [12,13], others indicate a higher risk of complications in DTI reconstructions [14].

Patient satisfaction and psychosocial outcomes are generally comparable between the two methods [15,16]. However, there remains a lack of comprehensive data on long-term aesthetic outcomes and on specific postoperative issues, such as AD.

AD is a significant aesthetic and functional concern in implant-based breast reconstruction. It is frequently observed in subpectoral reconstructions, where contraction of the pectoralis muscle results in visible implant movement or breast shape distortion [17,18,19]. Reported incidence rates in subpectoral techniques range from 75% to 100% [20,21]. Nevertheless, AD was also reported in prepectoral reconstructions [17,22]. AD not only affects the aesthetic outcome of the reconstruction but can also cause physical discomfort, pain and activity-related distress [22]. This may be particularly concerning in physically active women.

Despite its high prevalence and impact on quality of life, there is only few data investigating AD in DTI compared to TE reconstruction. To address this issue, validated assessment tools such as the BREAST-Q Animation Deformity Scale have recently been developed to assess patient-reported outcome measures (PROMs) and guide clinical decision-making [23]. A deeper understanding of AD is essential to refine surgical techniques and optimize reconstructive outcomes.

## 2. Materials and Methods

### 2.1. Study Design

This prospective, single-center cohort study included 67 patients who underwent implant-based breast reconstruction after nipple-sparing mastectomy (NSME) and skin-sparing mastectomy (SSME) due to breast cancer or prophylactic indications at the Department of Gynecology and Obstetrics and the Department of Plastic, Reconstructive, and Aesthetic Surgery at the Medical University of Innsbruck. Ethical approval was obtained from the Institutional Ethics Committee (protocol code 1327/2023). Two matched patient groups were evaluated: patients undergoing DTI versus TE reconstructions.

The primary endpoint of this study was the aesthetic outcome at long-term follow-up, assessed using the Breast Aesthetic Scale. Secondary endpoints included the prevalence and severity of animation deformity (AD), the Satisfaction with Breasts domain of the BREAST-Q questionnaire, the comparison of complication rates (Clavien Dindo 3a or 3b events), and the frequency of secondary (“touch up”) procedures between reconstruction techniques (DTI vs. TE).

Written informed consent was obtained from all participants for surgical procedures, photo documentation, anonymized evaluation, and publication of data. Inclusion criteria were defined as age > 18 years, breast reconstruction due to breast cancer or high-risk genetic predisposition, unilateral or bilateral reconstruction, and a postoperative follow-up period of at least 12 months. In the TE group, the follow-up period began on the date of the second-stage procedure (the exchange of the tissue expander for the definitive implant). Patients were excluded if they were lost to follow-up, deceased, underwent implant removal, declined participation in the clinical trial, had severe metastatic disease or psychiatric conditions, lacked standardized pre- or postoperative photographs, or had a follow-up period of less than 12 months. In the TE group, patients were excluded if the expander was not replaced with a definitive implant.

Demographic data collected included age, body mass index (BMI), smoking habits, and comorbidities (such as Type II diabetes mellitus and hypertension). Surgical technique, postoperative complications, and any secondary (“touch-up”) procedures were recorded.

Dynamic contour changes were assessed during clinical follow-up and by analyzing standardized photographs taken at rest and during upper-body movement. Changes associated with pectoralis contraction were classified as animation deformity (AD), while other movement-related deformities were recorded descriptively. Two established grading systems for AD were applied: the quantitative grading scale by Kim et al. [24] and the classification by Vidya et al. [25] All assessments were independently reviewed by a panel of three plastic surgeons with expertise in breast reconstruction. In cases of bilateral reconstruction, each breast was assessed independently.

To evaluate PROMs and quality of life, participants completed the validated German version of the postoperative BREAST-Q Reconstructive Module (Copyright ©2017, Memorial Sloan Kettering Cancer Center and the University of British Columbia) [23,26] once during their most recent follow-up visit. Scoring was done using the official conversion tables. Patients who did not respond to specific questionnaire items were excluded from analyses of those items but included in the overall analyses.

Aesthetic outcomes were independently assessed by a gender-balanced panel of four plastic surgeons (two senior consultants and two residents) using standardized postoperative photographs. Evaluations were conducted using the German-translated version of the validated Breast Aesthetic Scale [26].

### 2.2. Patients

All patients who underwent DTI and TE breast reconstruction at our department between 2009 and 2021 were considered for inclusion. During this period, 249 patients were identified. Of these, 14 patients passed away during follow-up, 73 patients were lost to follow-up, and 24 declined participation in the study. 10 patients were excluded due to advanced disease, and 26 lacked standardized pre- and/or postoperative photographic documentation. Implant removal had been performed in 25 patients; these cases were therefore excluded from the study.

Additionally, in the TE group, 10 patients did not proceed with treatment concerning the exchange from tissue expander to a definitive implant and were therefore excluded. Ultimately, 67 patients met the inclusion criteria and were enrolled in the study. All participants underwent primary implant-based breast reconstruction. Exclusion criteria and patient distribution across groups are summarized in Table 1.

### 2.3. Statistical Analysis

Statistical analysis was performed using ©Microsoft Excel (Microsoft Corporation, Washington, DC, USA) and ©SPSS Statistics Software (Version 30, IBM, New York, NY, USA). Chi-square tests, Fisher’s exact tests, and Student’s *t*-tests, as well as nonparametric comparisons, were used to assess differences between DTI versus TE groups and between prepectoral versus subpectoral implant placement. Pearson’s correlation coefficient was used to evaluate correlations. A *p*-value of less than 0.05 was considered statistically significant for all tests. Univariate linear regression was calculated to assess the potential confounding effects of age, BMI, radiation, chemotherapy, type of reconstruction, reconstruction side, and follow-up time. Variables that showed significance in univariate testing were entered into multiple regression models.

## 3. Results

### 3.1. Patient Characteristics

67 patients were included in this study, with 33 in the DTI group and 34 in the TE group. An overview of patient characteristics is provided in Table 2. The mean age was comparable between groups (DTI: 49.5 ± 12.6 years vs. TE: 52.3 ± 7.6 years; *p* = 0.38). Mean follow-up duration was 78.1 ± 45.4 months (approximately 6.5 years), and the mean BMI was 23.0 ± 3.8 kg/m^2^. BMI, mastectomy volume, and reconstruction volume did not differ significantly between groups. Comorbidities were similarly distributed, with no cases of diabetes in either group. Smoking and hypertension rates were slightly higher in the TE group, but without statistical significance. All reconstructions were performed primarily, and the proportion of unilateral versus bilateral procedures was comparable.

Most patients underwent nipple-sparing mastectomy (NSME, DTI: 88%, TE: 71%). Subpectoral implant placement was more common in both groups, though prepectoral placement was performed more frequently in the DTI group (DTI: 24% vs. TE: 9%). Rates of radiotherapy and chemotherapy (adjuvant and neoadjuvant) were comparable between groups. The primary indication for mastectomy was breast cancer (DTI: 68%, TE: 72%), with the remaining procedures performed prophylactically.

### 3.2. Complications & Secondary Corrections

Postoperative complications were classified according to the Clavien-Dindo Classification, graded as 3a or 3b, requiring surgical intervention under local or general anesthesia [27]. These included necrosectomy, hematoma evacuation, surgical infection control, and seroma evacuation. Overall, complications occurred in 6 patients (18.2%) in the DTI group and in 5 patients (14.7%) in the TE group. However, this difference was not statistically significant (*p* = 0.70). Specific postoperative events are summarized in Table 3.

Secondary corrective procedures were performed in 12 patients (36.4%) in the DTI group and in 13 patients (38.2%) in the TE group (*p* = 0.87). Implant exchange was the most common revision in the DTI group (30.3%) compared to 14.7% in the TE group. Nipple-areola-complex (NAC) reconstruction or areola tattooing was performed in 14.7% of TE patients but in none of the DTI cases. The indications for NAC reconstruction or areola tattooing were based on the SSME technique, not on NAC necrosis. Other revisions, such as lipofilling, capsulectomy, scar correction, and mastopexy, were infrequent and similarly distributed between groups. Secondary corrections are summarized in Table 4.

### 3.3. BREAST-Q Evaluation

All patients included in this study completed the BREAST-Q Reconstruction Module at least 12 months postoperatively. Details are presented in Table 5. The analysis revealed no statistically significant differences between the DTI and TE groups across any of the measured domains. Scores related to AD were similar between groups (DTI: 66.8 ± 21.6 vs. TE: 69.0 ± 21.6; *p* = 0.54), indicating comparable patient-perceived impact of AD regardless of reconstruction technique. Subscales assessing the adverse effects of radiation therapy showed generally low scores in both groups, with no significant differences.

### 3.4. Cosmetic Results

Regarding the overall appearance of reconstructive outcomes, both groups showed comparable results with a median of 3.5 points (*p* = 0.83). No significant differences were observed between groups with respect to breast symmetry, breast position, inframammary fold appearance, volume, shape and contour, scar appearance, as well as nipple position and symmetry. The detailed results are shown in Table 6. Bilateral reconstructions yielded significantly higher scores compared to unilateral reconstructions (*p* = 0.001), indicating a more favorable aesthetic result.

### 3.5. Animation Deformity (AD)

**Figure 1:**
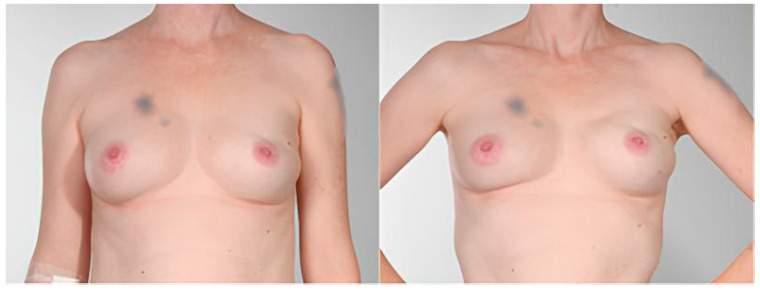
Left-sided prepectoral DTI breast reconstruction showing visible contour irregularities (Grade II Deformity according to Vidya Classification) at 5-year follow-up: Appearance at rest (left) and during movement (right). Patient tattoos above the right breast and left upper arm have been digitally blurred for anonymization.

The presence of AD was evaluated in all included patients using the scale by Kim et al. [24] and Vidya et al. [25] Breast animation deformity (Vidya grade II–IV) was clinically observed in 61.2% of patients, with significantly higher prevalence in the DTI group (*p* = 0.02). However, in our cohort, no significant correlation with implant placement was observed. Six cases of dynamic contour irregularities were identified in the prepectoral DTI group. Table 7 shows the number of AD cases by severity grade according to the rating scales described above, and Table 8 provides a descriptive summary of these patients. An illustrative case of Grade II deformity (Vidya classification) is presented in Figure 1.

Regarding patient-reported outcome measures (PROMs), assessment of AD using the validated BREAST-Q Animation Deformity Scale showed higher scores in patients with prepectoral implants compared to those with subpectoral reconstruction (mean score: prepectoral 79 vs. subpectoral 66). Although this trend suggests a favorable outcome for the prepectoral approach, the difference was not statistically significant (*p* = 0.21).

### 3.6. Confounding Factors

In univariate linear regressions, age and follow-up time were identified as significant confounders for the Breast Aesthetic Scale score. In the multivariate model, only age remained significant. For Clavien–Dindo complication grading, age and bilateral surgery were identified as potential confounders, but did not remain significant in the multivariate model.

## 4. Discussion

In this prospective cohort study comparing DTI and TE breast reconstruction outcomes, no significant differences in long-term PROMs were observed, as shown by the BREAST-Q questionnaire scores. These findings align with previous studies suggesting that both techniques can provide satisfactory, comparable quality-of-life outcomes following mastectomy [12,15]. A 2023 meta-analysis demonstrated that DTI breast reconstruction yields patient satisfaction and quality of life outcomes similar to or even superior to those of autologous tissue transfer when both autologous and implant-based breast reconstruction options are available [28].

This study also found no difference in patient-perceived impact of AD, a noteworthy concern in implant-based breast reconstruction. While AD has traditionally been associated with subpectoral placement [17], our findings indicate that subjective experiences of AD, as measured by the BREAST-Q Animation Deformity Scale, did not differ significantly between DTI and TE groups.

Recent literature has introduced innovative surgical techniques for managing AD and reported excellent postoperative BREAST-Q Scores for “overall satisfaction with outcome” [18]. However, the BREAST-Q Animation Deformity Scale [23], which allows for a more specific and standardized evaluation of animation-related symptoms and their impact on patient-reported outcomes, was not used in this context.

As expected, patients who underwent prepectoral implant-based reconstruction reached higher scores on the BREAST-Q Animation Deformity Scale—indicating greater satisfaction—than those with subpectoral placement. This finding aligns with the existing literature, which suggests that avoiding muscle dissection in prepectoral reconstruction reduces animation deformity [17,29]. However, in our cohort, the difference did not reach statistical significance (mean score: prepectoral 79 vs. subpectoral 66; *p* = 0.209). This indicates that while prepectoral placement tends to be associated with less visible or bothersome deformity during muscle contraction, some degree of deformity may still be present and perceptible to patients. This underscores the subjective nature of this outcome and highlights the importance of patient-reported satisfaction when evaluating reconstructive techniques. Further studies with a larger sample size may help to clarify the clinical relevance of this trend.

AD is most frequently observed in subpectoral implant placement [17]. However, some patients with prepectoral reconstructions report visual irregularities or implant movement during certain activities, which may be mistaken for AD. This phenomenon, however, lacks the muscular etiology characteristic of true animation deformity. To date, there is no established term to describe this phenomenon.

**Figure 2:**
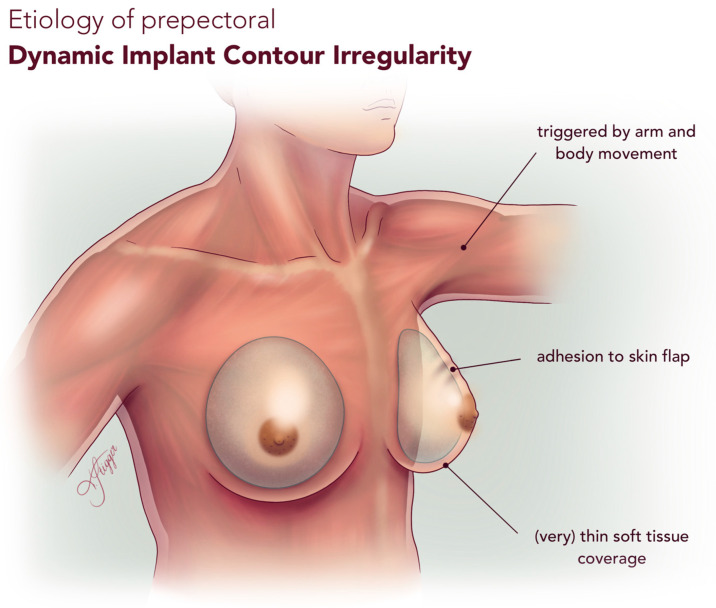
Etiology of prepectoral Dynamic Implant Contour Irregularity (DICI).

We propose the term *Dynamic Implant Contour Irregularity (DICI)* to define this distinct entity. DICI may result from factors such as implant adhesion to the mastectomy skin flap, implant mobility within the pocket, or thin soft tissue coverage. It may mimic AD or capsular contracture (CC), leading to diagnostic or terminological confusion. In contrast to CC [30], which is static and often associated with pain, DICI is primarily a dynamic phenomenon in which pain appears to be uncommon; unlike true AD, DICI is not reproducible with pectoralis muscle contracture, but with arm and upper body movement. A comparison between these three distinct entities is presented in Table 9, and the etiology of DICI is visualized in Figure 2.

In our cohort, we observed DICI in six patients who underwent DTI reconstruction with prepectoral implant placement. Notably, the patient population in this study was mostly lean with a mean BMI of 23.0 ± 3.8 kg/m^2^. This relatively low BMI may partially account for the higher-than-expected incidence of contour irregularities in prepectoral implant reconstructions, as reduced soft tissue coverage in thinner patients likely increases the visibility of implant-related surface changes. Although animation deformity appeared more frequent in the DTI group, the limited number of prepectoral cases restricts statistical power. Therefore, the absence of correlation with implant plane should be interpreted with caution. The prevalence of DICI in the DTI subgroup may be related to the absence of a progressive tissue expansion phase, which, in TE reconstruction, allows gradual adaptation and thickening of the overlying soft tissue envelope before final implant placement.

Management of AD is well studied and may offer valuable insights into therapeutic strategies for DICI. One of the most effective methods for managing AD is converting from subpectoral to prepectoral implant placement, which eliminates the deforming influence of the pectoralis muscle. Several studies have reported favorable outcomes with this technique [31,32,33]. Adjunctive procedures, such as the use of acellular dermal matrix (ADM) and autologous fat grafting, can further improve contour by camouflaging visible implant-related deformities and reducing rippling, particularly in patients with thin mastectomy skin flaps [31,34].

These strategies—fat grafting and pocket modifications in particular—may be extrapolated for the treatment of DICI. Different from AD in its mechanism and presentation, DICI shares clinical features such as dynamic visibility and contour irregularities.

Given the complexity of dynamic contour irregularities—whether due to muscular animation or surface-level implant deformities—standardized assessment tools are essential to improve diagnosis, comparison, and treatment evaluation. In this context, grading scales such as the Kim and Vidya systems have emerged as crucial instruments for evaluating AD, though each has its own strengths and limitations.

The Kim Scale is based on video documentation and offers an objective, reproducible method for grading AD. It aims to quantify visible implant movement during muscle contraction and ranges from Grade I to III [24]. However, a key limitation is the absence of Grade 0, which would represent the complete absence of AD. This can distort comparisons between patient groups and underrepresent the baseline population without AD.

In contrast, the Vidya Scale offers a broader assessment range from grade 1 to 4, with grade 1 indicating no signs of AD. Grades 2 to 4 show increasing severity of deformity. Notably, the Vidya scale includes the patient’s perspective, which is essential when evaluating subjective aesthetic concerns.

To comprehensively evaluate surface-level changes after implant-based breast reconstruction, objective observations (such as video analysis) and subjective patient feedback are required. The concept of DICI remains exploratory and is based on a limited number of cases. To build on these preliminary findings, we plan a prospective study with a contemporary cohort and a larger sample size to further investigate implant-related motion artifacts, such as DICI and AD, using standardized video documentation. Future studies should also include interobserver validation and combine objective grading with patient-reported outcomes to establish reproducible, clinically relevant diagnostic criteria.

In our cohort, there was a trend toward greater reconstruction volume in the DTI group than in the TE group (*p* = 0.06), which may reflect the selection of larger implants in direct-to-implant procedures. While not statistically significant, this difference could have contributed to subtle variations in postoperative contour and aesthetic outcomes.

This study benefits from a prospective design, long-term follow-up, and the use of validated standardized tools (BREAST-Q, Breast Aesthetic Scale), offering valuable clinical insights into long-term aesthetic outcomes and PROMs. However, several limitations need to be acknowledged. First, the relatively small sample size and single-center setting may restrict the generalizability of the findings. The reduced final sample size and the exclusion of cases with explantation or incomplete reconstruction may introduce selection bias, as these cases may reflect adverse outcomes. Nonetheless, their exclusion was necessary to ensure standardized comparison of long-term results. Future multicenter studies including such cases are warranted. Only 11 of 67 patients underwent prepectoral implant placement, compared with 56 who received subpectoral reconstruction. Furthermore, only a small portion of patients (6 of 67) received radiotherapy, as we favored immediate autologous breast reconstruction in cases where radiotherapy was planned. This reflects the clinical practice at the time, where patients requiring radiotherapy were mainly treated with autologous breast reconstruction, thereby reducing the representativeness of irradiated implant-based cases in our cohort. Another limitation is reliance on clinical observation rather than objective imaging to evaluate aesthetic outcomes and contour irregularities, which may compromise the precision of outcome assessment. Finally, the patient cohort reflects surgical practice from 2009 to 2021—since then, reconstructive trends have increasingly shifted toward prepectoral implant placement, also at our department. To address this, further evaluation of a contemporary cohort is planned to generate more balanced and up-to-date insights into implant-based breast reconstruction.

## 5. Conclusions

Our findings demonstrate that both DTI and TE breast reconstruction techniques yield comparable long-term outcomes in terms of patient satisfaction and overall aesthetic outcomes. The data suggest that the choice between DTI and TE reconstruction can be guided by individual clinical considerations and patient preferences, without affecting long-term quality of life or cosmetic results. Notably, bilateral reconstruction was associated with significantly higher aesthetic ratings, highlighting the importance of symmetry in achieving favorable aesthetic outcomes.

Breast animation deformity was observed frequently, particularly among DTI patients. While no significant correlation with implant placement (subpectoral vs. prepectoral) could be demonstrated in this cohort, the limited number of prepectoral reconstructions warrants cautious interpretation. Additional factors, including surgical technique, muscle dynamics, and the individual anatomy, may contribute to the observed variation in dynamic implant-related contour changes. Moreover, the identification of dynamic implant contour irregularities in a subset of patients with prepectoral implant placement highlights the need to broaden the current understanding of implant-related motion artifacts. These observations point to the limitations of the traditional definition of breast animation deformity and support the introduction of a more nuanced classification system for dynamic implant deformations.

Overall, this study underscores the importance of long-term follow-up and comprehensive evaluation—including both surgeon-assessed and patient-reported outcomes—to optimize surgical planning and refine the criteria for procedural selection in implant-based breast reconstruction.

## Figures and Tables

**Table 1:** Exclusion Criteria.

	Direct-to-Implant (DTI) n = 128	Tissue Expander (TE) n = 121	Total n = 249
Deceased	7	7	14
Lost to Follow-up	37	36	73
Participation declined	16	8	24
Severe Disease	9	1	10
Lack of photographic Documentation	16	10	26
Loss of Implant	10	15	25
TE: no Exchange of TE for final Implant	-	10	10
**Participants**	**33**	**34**	**67**

n = patients.

**Table 2:** Patient Characteristics.

	Direct-to-Implant (DTI) * n = 33		Tissue Expander (TE) * n = 34		
	MEAN	SD	MEAN	SD	*p*-Value
Age (years)	49.50	12.6	52.28	7.61	0.38
Follow-up (months)	87.22	43.78	69.44	45.76	**0.03**
BMI (kg/m^2^)	22.61	2.73	23.29	4.64	0.89
Mastectomy Volume (g)	274.71	169.2	227.19	237.75	0.22
Reconstruction Volume (g)	267.96	114.29	332.19	115.73	0.06
Expander Filling at Time of Surgery (mL)			144.6	136.1	
	n	%	n	%	
Active smoker *	6	18.18	7	20.59	0.76
Hypertension *	5	15.15	7	20.59	0.75
Diabetes mellitus Type II *	0	0	0	0	
Mastectomy *					0.08
- SSME	4	12.12	10	29.41	
- NSME	29	87.88	24	70.59	
Reconstruction Side *					0.81
- Unilateral	19	57.58	21	61.76	
- Bilateral	14	42.42	13	38.24	
Implant Plane *					0.08
- Prepectoral	8	24.24	3	8.82	
- Subpectoral	25	75.76	31	91.18	
Radiotherapy *					1.0
- Yes	3	9.09	3	8.82	
- No	30	90.91	31	91.18	
Chemotherapy *					0.36
- Yes	12	36.36	5	14.71	
- Adjuvant	5	15.15	6	17.65	
- Neoadjuvant	7	21.21	3	8.82	
- No	21	63.64	25	73.53	
Indication for Mastectomy ^♦^					0.76
- Breast Cancer	32	68.09	34	72.34	
- Prophylactic	15	31.91	13	27.66	

* n = patients, ^♦^ n = breasts.

**Table 3:** Complications (Clavien-Dindo 3a and 3b).

	Direct-to-Implant (DTI) * n = 33		Tissue Expander (TE) * n = 34		
	* n	%	* n	%	*p*-Value
Secondary Wound Revision	1	3.03	2	5.88	0.97
Necrosectomy	3	9.09	2	5.88	
Haematoma Evacuation	2	6.06	2	5.88	
Surgical Infection Control	1	3.03	0	0	
Seroma Evacuation	1	3.03	1	2.94	

* n = patients.

**Table 4:** Secondary Corrections.

	Direct-to-Implant (DTI) * n = 33		Tissue Expander (TE) * n = 34		
	* n	%	* n	%	*p*-Value
NAC Reconstruction/Areola Tattoo	0	0	5	14.71	0.57
Lipofilling	2	6.06	4	11.76	
Capsulectomy	1	3.03	1	2.94	
Implant Exchange	10	30.30	5	14.71	
Scar Correction	1	3.03	2	5.88	
Mastopexy	1	3.03	2	5.88	

* n = patients; NAC—nipple-areola-complex.

**Table 5:** BREAST-Q Scores.

	Direct-to-Implant (DTI) * n = 33		Tissue Expander (TE) * n = 34		
	MEAN	SD	MEAN	SD	*p*-Value
Psychosocial Well-Being	74.70	19.45	71.94	17.43	0.71
Sexual Well-Being	58.31	22.29	58.56	23.81	0.79
Satisfaction with Breasts	63.97	19.17	61.70	15.45	0.97
Satisfaction with Implants (a)	2.94	1.01	3.21	0.78	0.34
Satisfaction with Implants (b)	3.03	0.97	3.21	0.82	0.51
Physical Well-Being Chest	71.00	22.49	75.76	12.51	0.57
Breast Animation Deformity	66.78	21.61	68.97	21.55	0.54
Adverse Effects of Radiation a	1.75	0.96	1.43	0.79	0.65
Adverse Effects of Radiation b	1.25	0.50	1.29	0.76	0.93
Adverse Effects of Radiation c	1.75	0.96	1.33	0.52	0.61
Adverse Effects of Radiation d	1.25	0.50	1.43	0.53	0.65
Adverse Effects of Radiation e	1.25	0.50	1.71	0.76	0.41
Adverse Effects of Radiation f	1.00	0.00	1.29	0.49	0.53
Satisfaction with Information	77.59	18.29	72.74	17.96	0.24
Satisfaction with Surgeon	88.55	21.33	93.18	14.99	0.21
Satisfaction with Medical Team	95.24	12.97	95.26	11.83	0.84
Satisfaction with Office Staff	97.26	7.38	95.21	11.76	0.99

* n = patients. Satisfaction with Implants a = satisfaction with the visual appearance of implant folds, b = satisfaction with tactile perception of implant folds.

**Table 6:** Breast Aesthetic Scale.

	Direct-to-Implant (DTI) ^♦^ n = 47			Tissue Expander (TE) ^♦^ n = 47			
	MEDIAN	Q_1_	Q_3_	MEDIAN	Q_1_	Q_3_	*p*-Value
Breast Symmetry	3.50	2.50	4.00	3.25	2.50	4.00	0.51
Breast Position	3.50	3.00	4.00	3.50	3.00	4.00	0.53
Inframammary Fold	4.00	3.00	4.00	4.00	3.00	4.00	0.38
Volume	3.50	2.50	4.00	3.75	3.00	4.00	0.90
Shape and Contour	3.00	2.00	4.00	3.00	2.50	3.50	0.67
Scar							
Appearance	4.00	3.50	5.00	4.00	3.00	4.00	0.31
NAC							
Nipple Symmetry	3.50	2.50	4.00	3.50	3.00	4.00	0.84
Nipple Position	3.00	2.50	4.00	3.50	2.50	4.00	0.65
Overall Appearance	3.50	2.50	4.00	3.50	2.50	4.00	0.83

**^♦^** n = breasts; NAC—nipple-areola-complex.

**Table 7:** Clinical Assessment of AD (Classification by Kim et al. [19] and Vidya et al. [20]).

		Direct-to-Implant (DTI) * n = 33		Tissue Expander (TE) * n = 34	
Classification	Definition	Subpectoral	Prepectoral	Subpectoral	Prepectoral
KIM [19]					
Grade I	*NAC dislocation <2 cm and contour irregularity <25% of breast surface*	11	7	20	3
Grade II	*NAC dislocation ≥2 cm and contour irregularity <25% of breast surface* *NAC dislocation <2 cm and contour irregularity ≥25% of breast surface*	11	0	10	0
Grade III	*NAC dislocation >2 cm and contour irregularity ≥25% of breast surface*	3	1	1	0
VIDYA [20]					
Grade I	*No visible distortion and displacement of implant*	6	2	15	3
Grade II	*Minimally visible distortion with displacement of implant; unnoticed by patient*	7	4	6	0
Grade III	*Moderately visible distortion with displacement of implant; noticed by patient*	10	2	9	0
Grade IV	*Severe distortion and persistent displacement of implant; disturbing for patient*	2	0	1	0

* n = patients; AD—animation deformity, NAC—nipple areola complex.

**Table 8:** Case description of patients showing DICI.

Pat.	Age (Years)	BMI (kg/m^2^)	NSME vs. SSME	DTI vs. TE	Implant Plane	Reconstruction Side	Reconstruction Volume (g)	Radiotherapy	Clavien Dindo
**A**	50	20.6	NSME	DTI	prepectoral	unilateral	120	no	0
**B**	36	18.7	NSME	DTI	prepectoral	unilateral	295	no	0
**C**	65	25.3	NSME	DTI	prepectoral	unilateral	560	no	3b
**D**	43	26.5	NSME	DTI	prepectoral	bilateral	350	yes	3b
**E**	57	24.0	NSME	DTI	prepectoral	bilateral	365	no	0
**F**	48	22.6	NSME	DTI	prepectoral	unilateral	235	no	0

Pat.—patient, BMI—body mass index, NSME—nipple sparing mastectomy, SSME—skin sparing mastectomy, DTI—direct to implant, TE—tissue expander.

**Table 9:** Comparison of DICI, AD, and CC.

Feature	DICI	AD	CC
**Deformity Type**	Implant displacement (any direction) Breast distortion	Implant displacement (superior or lateral) Breast distortion	Implant distortion (any direction) Hardening Asymmetry
**Etiology**	Implant adhesion to mastectomy skin flap Implant mobility during arm and body movement (Very) thin mastectomy skin flap	Pectoralis muscle contraction	Fibrotic capsule tightening around implant
**Implant Placement**	Prepectoral	Subpectoral	Either
**Trigger**	Arm/body movement	Pectoralis muscle contraction	None (static deformity)
**Associated Pain**	Rare	Rare	May be present
**Diagnosis**	Clinical observation Reproducible with motion, not necessarily by pectoralis muscle contraction	Clinical observation Reproducible with pectoralis muscle contraction	Clinical observation Baker grade (I-IV) Imaging
**Management**	Fat grafting Pocket revision	Conversion to prepectoral implant placement Muscle detachment	Capsulotomy/Capsulectomy Implant explantation/exchange

DICI—dynamic implant contour irregularity, AD—animation deformity, CC—capsular contracture.

## Data Availability

The data presented in this study are available on request from the corresponding author.

## Abbreviations

The following abbreviations are used in this manuscript:

AD	animation deformity
ADM	acellular dermal matrix
CC	capsular contracture
DICI	dynamic implant contour irregularity
DTI	direct-to-implant
NAC	nipple-areola-complex
NSME	nipple-sparing mastectomy
PROM	patient-reported outcome measure
SSME	skin-sparing mastectomy
TE	tissue expander
